# Women Are Also Disadvantaged in Accessing Transplant Outside the United States: Analysis of the Spanish Liver Transplantation Registry

**DOI:** 10.3389/ti.2024.12732

**Published:** 2024-05-07

**Authors:** Marta Tejedor, Fernando Neria, Gloria De La Rosa, Carolina Almohalla Álvarez, María Padilla, Andrea Boscà Robledo, Yiliam Fundora Suárez, Francisco Sánchez-Bueno, Miguel Angel Gómez-Bravo, Marina Berenguer

**Affiliations:** ^1^ Infanta Elena University Hospital, Valdemoro, Spain; ^2^ Universidad Francisco de Vitoria, Pozuelo de Alarcón, Spain; ^3^ National Transplant Organization, Madrid, Spain; ^4^ Scientific Committee of the National Liver Transplant Registry, Madrid, Spain; ^5^ La Fe Hospital, Valencia, Spain; ^6^ Hospital Clinic of Barcelona, Barcelona, Spain; ^7^ Virgen de la Arrixaca University Hospital, Murcia, Spain; ^8^ Virgen del Rocío University Hospital, Seville, Spain; ^9^ Hepatology—Liver Transplantation Unit, IIS La Fe and CIBER-EHD, Hospital Universitari i Politècnic La Fe, Valencia, Spain; ^10^ Department of Medicine, Universitat de València, Valencia, Spain

**Keywords:** sex inequity, waiting list, survival, access to transplantation, women, Spanish Liver Transplant Registry (RETH)

## Abstract

Sex inequities in liver transplantation (LT) have been documented in several, mostly US-based, studies. Our aim was to describe sex-related differences in access to LT in a system with short waiting times. All adult patients registered in the RETH-Spanish Liver Transplant Registry (2000–2022) for LT were included. Baseline demographics, presence of hepatocellular carcinoma, cause and severity of liver disease, time on the waiting list (WL), access to transplantation, and reasons for removal from the WL were assessed. 14,385 patients were analysed (77% men, 56.2 ± 8.7 years). Model for end-stage liver disease (MELD) score was reported for 5,475 patients (mean value: 16.6 ± 5.7). Women were less likely to receive a transplant than men (OR 0.78, 95% CI 0.63, 0.97) with a trend to a higher risk of exclusion for deterioration (HR 1.17, 95% CI 0.99, 1.38), despite similar disease severity. Women waited longer on the WL (198.6 ± 338.9 vs. 173.3 ± 285.5 days, *p* < 0.001). Recently, women’s risk of dropout has reduced, concomitantly with shorter WL times. Even in countries with short waiting times, women are disadvantaged in LT. Policies directed at optimizing the whole LT network should be encouraged to guarantee a fair and equal access of all patients to this life saving resource.

## Graphical Abstract

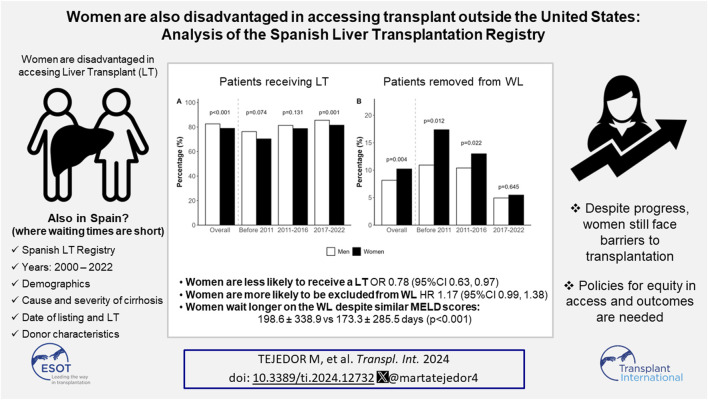

## Introduction

In recent years, noticeable health disparities between men and women have emerged, extending into various domains, including the transplant arena. Indeed, although sex differences exist from biological and physio-pathological perspectives, these have rarely been considered when proposing prognostic models or when applying and evaluating treatments. Because the demand for organs has always exceeded the supply, the transplant community has long recognized the need to ensure equity and efficiency of the organ allocation system. With this in mind, it is imperative to recognize inequities to then further develop policies that have the potential to ensure that women have equitable access to transplantation. In that sense, providing national data is crucial as poorer access to liver transplantation (LT) for women compared to men might be explained by different analytical approaches or different national contexts, and has two facets, biological and sociocultural [1, 2]. Sex inequities in LT including the type of liver disease that leads to the need of transplantation, the referral pattern to transplant centres, access to waiting lists (WL) and transplantation itself as well as post-transplant outcomes have been recently documented in several, mostly US-based, studies [1–5]. The reduced need of LT, mainly explained by the different prevalence of chronic liver disease in women and men, particularly refers to viral cirrhosis and liver cancer, more frequently found in men [1–4, 6]. However recent changes in epidemiology due to the advent and penetration of direct antiviral agents as well as the obesity epidemics can modify this scenario and are known to vary substantially based on local epidemiology [7, 8]. Several hypotheses attempt to elucidate the higher likelihood of death on the WL, removal from the list due to an illness precluding transplant, and the lower likelihood of receiving a liver graft. Factors such as limitations in the model for end-stage liver disease (MELD) score and donor-recipient size mismatch are implicated [9–15], and these variables strongly correlate with local allocation systems and general characteristics of the local population. In summary, our transplant population (including transplant candidates and recipients) may have substantial differences from that of the US, related to both transplant indications as well as baseline features of the population.

The so called “Spanish Model in Organ Donation and Transplantation” has positioned our country as a global leader in terms of donation and transplantation. The key features of this model include its three-tiered governing structure, close and collaborative relationships with the media, dedicated professional roles, a comprehensive reimbursement strategy, and intensive tailored training programs for all personnel. Throughout the years, the pool of donors has expanded, with a significant rise in donation after circulatory death (DCD). The program is driven by a culture of research, innovation, and continuous commitment and is complemented by successful strategies in prevention of end-stage liver and renal disease [16, 17]. As in most Eurotransplant countries, exception points are assigned to some indications where WL mortality risk is not accurately predicted by MELD, particularly hepatocellular carcinoma (HCC). The registered MELD scores for HCC patients have been adjusted over time to facilitate access to LT while avoiding disadvantages for non-HCC patients. Overall, patients listed for HCC can be registered at a MELD score equivalent to a 15% probability of patient death within 3 months and upgraded every 90 days to a MELD score that reflects an increase in mortality by 10% [18].

The MELD system was progressively adopted in different regions of Spain since 2003 becoming the allocation method of choice in most of the country in 2011. Previously, a combination of time on WL and Child-Pugh score were used to allocate organs.

The aim of our study was to describe the recipient profile over time in Spain, particularly with regards to potential sex-related differences in access to LT in a system with short waiting times.

## Material and Methods

### Study Population

All adult (18 years old or older) patients registered in the Spanish Registry for Donation and Transplantation (CORE), managed by the Organización Nacional de Trasplantes (ONT), from 2000 to 2022 were included in this study. Urgent transplants, due to acute or subacute liver failure, were excluded as the criteria to allocate this group differs significantly from those with chronic end-stage liver disease [19, 20]. Combined transplants were also excluded as the concurrence of extra-hepatic organ failure requiring transplantation may influence waiting times and may require non-standard exception points or specific organ allocation policies [21–23]. We also excluded re-transplants, as standard allocation systems may not apply in all the Spanish system. Registrants were followed from the time of inclusion on the WL until the 31st of December 2022, LT, removal from the list or death, whichever occurred first. Reasons for removal included being too sick for transplantation or improvement such that LT was no longer needed, although our analysis focused on patients excluded for deterioration or death.

Variables analysed were: baseline demographics (age, sex, blood group, weight and height), presence of HCC, cause and severity of liver disease resulting in end-stage liver disease, date of listing on the LT WL and date of transplantation. Donor baseline characteristics were also analysed: age, sex, weight, height, and type of donation [donation after brain death (DBD), DCD, living donation (LD), domino].

Three time periods were analyzed: from 2000 to 2010, from 2011 to 2016, and from 2017 to 2022. Since MELD was adopted by most of the country as the preferred allocation system from 2011, this date was chosen for the first cut-off. The remaining time was divided into two equally long periods to assess the evolution of the WL.

This research was conducted in accordance with both the Declarations of Helsinki and Istanbul. We retrospectively explored data collected from the Spanish Liver Transplant Registry (Registro Español de TrasplanteHepático, RETH). RETH is a multicenter registry that recruits data from all liver transplant units in Spain with periodic auditing. This study was based on data routinely collected at a national level for organ allocation and to assess the efficacy and safety of the LT program. For that reason, the requirement for a formal ethics committee review was waived by the National Transplant Organization (Organización Nacional de Trasplante, ONT). The data analyzed in this study is subject to the following licenses/restrictions: datasets belong to Spanish Liver Transplant Society and are managed and administered by the National Transplant Organization.^1^

### Statistical Analysis

Continuous variables are expressed as mean and standard deviations. T-test or ANOVA test were used as appropriate. Categorical variables were compared using the Chi-square test when appropriate. A multiple regression analysis was performed to assess transplantation odds ratio (OR). A Cox proportional hazards multiple regression analysis was performed to determine whether sex was associated with the likelihood of removal from the list due to worsening or death; this approach was used to account for differences in follow-up times after inclusion in WL. All analyses were stratified by sex and adjusted where appropriate by age, blood group and height, and MELD when available, at time of LT. A *p*-value <0.05 was considered statistically significant. Sub-analyses were performed in case missing information was significant for a specific variable (i.e., MELD). All statistical analyses were performed with the software R version 4.2.3.

## Results

### Baseline Characteristics of Patients on the WL

**FIGURE 1:**
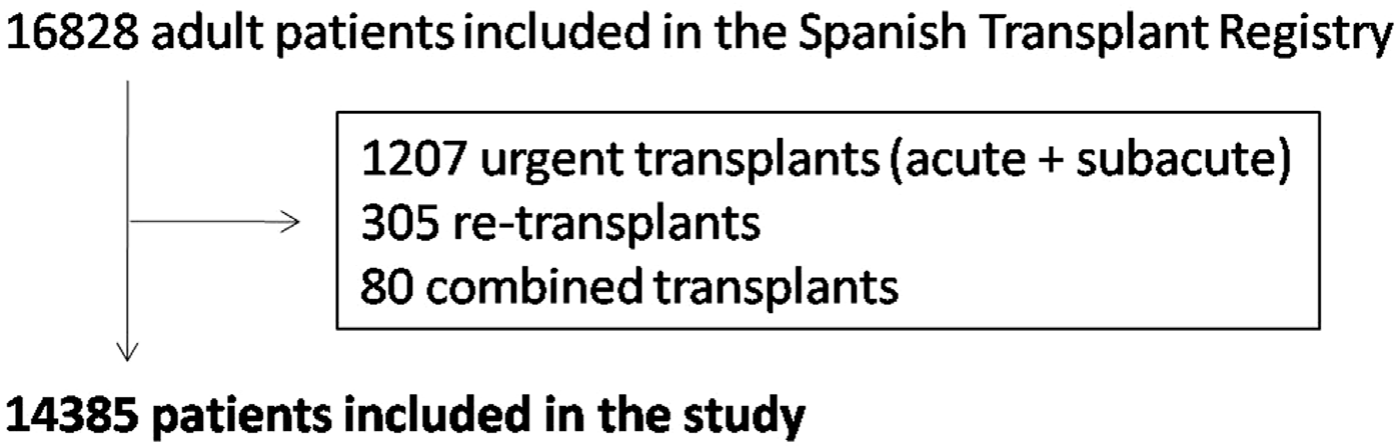
Flowchart of the study.

**FIGURE 2:**
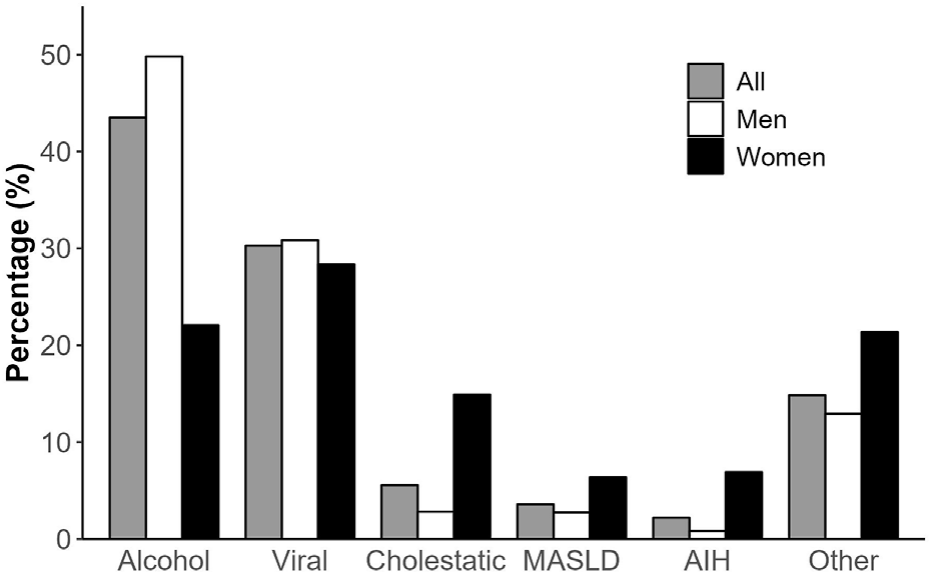
Aetiology of liver disease, overall and by sex. MASLD, metabolic dysfunction-associated steatotic liver disease; AIH, autoimmune hepatitis.

Out of 16,828 adult patients included in the CORE registry, a total of 14,385 patients meeting the inclusion criteria were analysed (Figure 1). Baseline characteristics of those included vs. those excluded are shown in Supplementary Table S1. Most listed patients were men (77%). Differences between included men and women are shown in Table 1 and Figure 2. As expected, several significant differences were observed by sex. In particular, men were older, heavier and taller. They suffered more of alcohol-related liver disease and HCC than women, who were more likely affected by cholestatic and autoimmune liver diseases.

**TABLE 1:** Liver transplant candidates baseline demographics, overall and by sex.

Variable	Overall (*n* = 14,385)	Men (*n* = 11,115)	Women (*n* = 3,270)	P^a^
Age (years)	56.2 ± 8.7	56.5 ± 8.2	55.5 ± 10.2	**<0.001**
Weight (kg)	77.3 ± 15.7	80.6 ± 14.7	66.1 ± 13.7	**<0.001**
Height (cm)	168.4 ± 8.6	171.0 ± 7.1	159.2 ± 7.0	**<0.001**
MELD^b^	16.6 ± 5.7	16.6 ± 5.7	16.6 ± 5.7	0.953
Blood group				**0.010**
· A	6,540 (45.5%)	5,084 (45.7%)	1,456 (44.5%)	
· O	5,872 (40.8%)	4,464 (40.2%)	1,408 (43.1%)	
· B	1,380 (9.6%)	1,094 (9.8%)	286 (8.8%)	
· AB	593 (4.1%)	473 (4.3%)	120 (3.7%)	
Aetiology				**<0.001**
· Alcohol	6,260 (43.5%)	5,538 (49.8%)	722 (22.1%)	
· Viral	4,356 (30.3%)	3,429 (30.9%)	927 (28.4%)	
· Cholestatic	801 (5.6%)	313 (2.8%)	488 (14.9%)	
· MASLD	514 (3.6%)	306 (2.8%)	208 (6.4%)	
· AIH	317 (2.2%)	91 (0.8%)	226 (6.9%)	
· Other	2,137 (24.8%)	1,438 (12.9%)	699 (21.3%)	
HCC	4,937 (34.3%)	4,230 (38.1%)	707 (21.6%)	**<0.001**

Continuous variables are expressed as Mean ± SD; categorical variables are expressed as n (%).

^a^ Welch Two Sample t-test for comparison between men and women (continuous variables); Pearson’s Chi-squared test (categorical variables).

^b^ MELD data only available for 5,475 patients. MASLD, metabolic dysfunction-associated steatotic liver disease; AIH, autoimmune hepatitis; HCC, hepatocellular carcinoma.

The bold values represent p values that are significant statistically.

### Evolution of the WL

**FIGURE 3:**
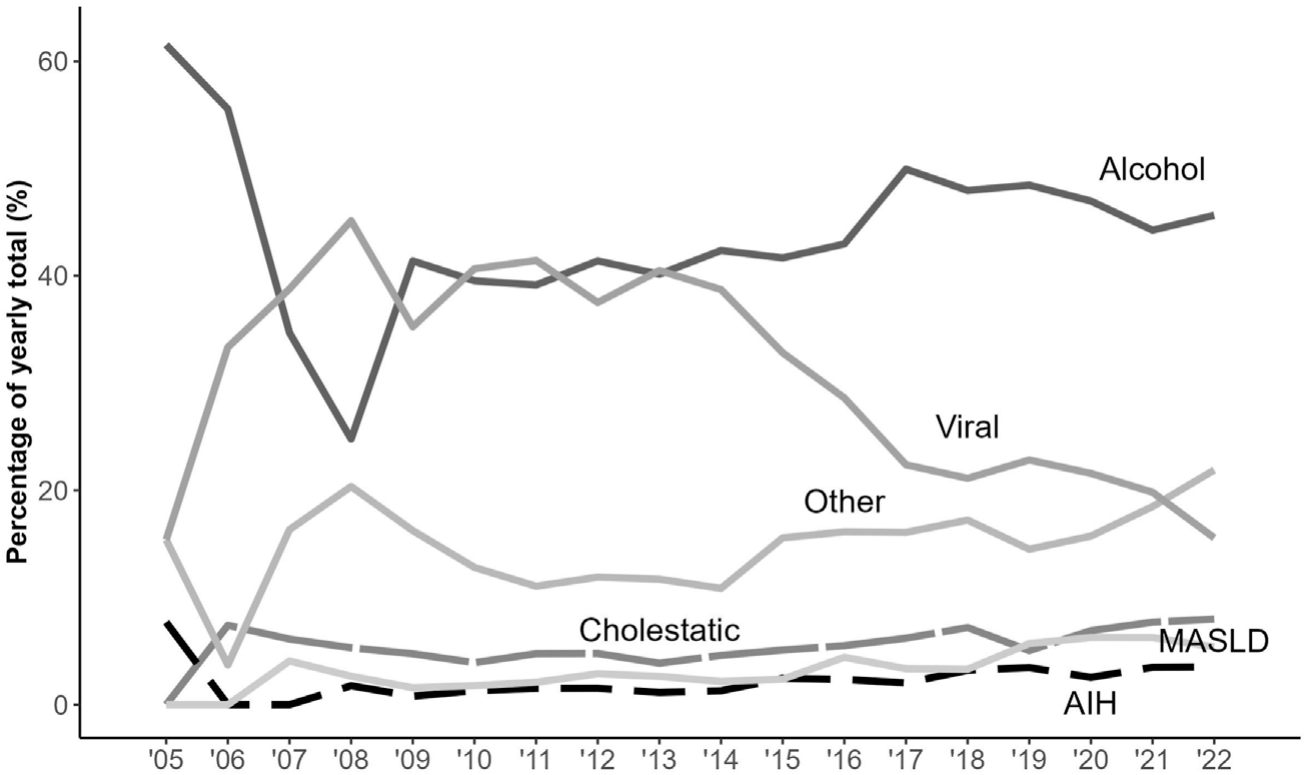
Changes in aetiology of liver disease over time. MASLD, metabolic dysfunction-associated steatotic liver disease; AIH, autoimmune hepatitis.

A change in the WL was observed over time; with candidates becoming older and heavier (Table 2). Alcohol-related liver disease and metabolic dysfunction-associated steatotic liver disease (MASLD) have become more frequent indications for LT, as opposed to a decrease in viral hepatitis (Figure 3).

**TABLE 2:** Evolution of the wait list (WL) demographics by period.

Variable	2000–2010 (*n* = 1,786)	2011–2016 (*n* = 6,640)	2017–2022 (*n* = 5,959)
Age (years)	53.9 ± 8.7	55.5 ± 8.6	57.8 ± 8.5
Weight (kg)	75.5 ± 14.8	77.1 ± 15.4	78.1 ± 16.1
Height (cm)	167.7 ± 8.8	168.5 ± 8.7	168.4 ± 8.6
MELD^a^	19.2 ± 5.2	16.8 ± 5.7	16.2 ± 5.8
Blood group			
· A	824 (46.1%)	3,031 (45.7%)	2,685 (45.1%)
· O	767 (43.0%)	2,687 (40.5%)	2,418 (40.6%)
· B	136 (7.6%)	643 (9.7%)	601 (10.1%)
· AB	59 (3.3%)	279 (4.2%)	255 (4.3%)
HCC	523 (29.3%)	2,373 (35.7%)	2,041 (34.3%)

Continuous variables are expressed as Mean ± SD; categorical variables are expressed as n (%). One-way ANOVA *p* < 0.05 for each variable between periods.

^a^ MELD data only available for 5,475 patients. HCC, hepatocellular carcinoma.

**FIGURE 4:**
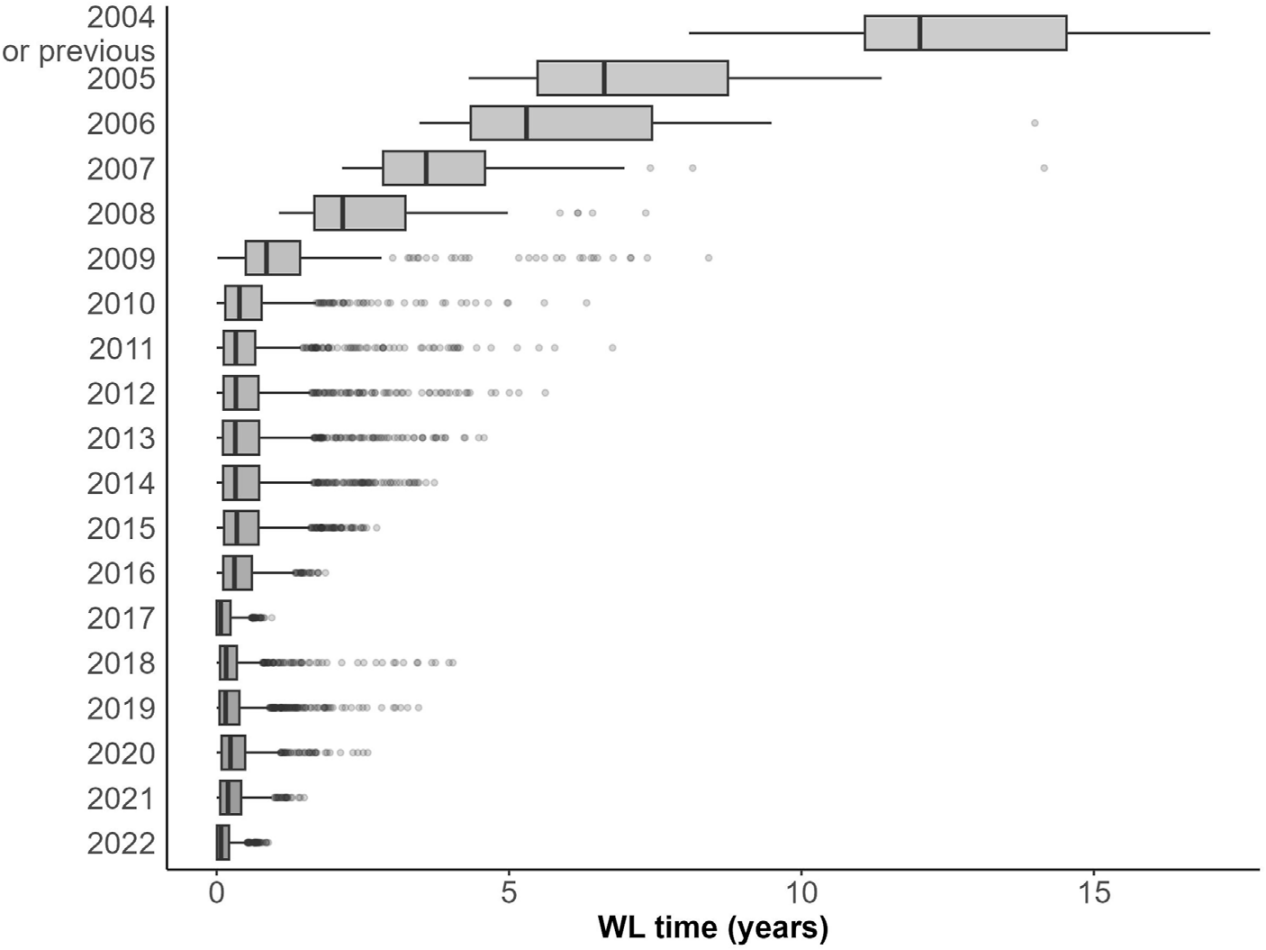
Evolution of waiting times over time.

Time on the WL has shortened from 424.3 ± 619.6 days in the first period (2000–2010), to 190.9 ± 229.6 days in the second period (2011–2016) and to 92.3 ± 126.0 days in the third period (2017–2022) (*p* < 0.001) (Figure 4). The progressive shortening of waiting times coincided with a progressive increase in the likelihood of receiving a transplant: compared to the first period, HR was 1.97 (95% CI 1.84, 2.11; *p* < 0.001) in the second period and 3.99 (95% CI 3.72, 4.28; *p* < 0.001) in the third period.

MELD was recorded in a non-systematic way in the national database from 2011 and was available for 5,350 of the 12,599 included patients (43%) after 2011. To ensure that all patients included in the WL after this date were comparable, differences between patients with available and unavailable MELD score were analysed and are shown in Supplementary Table S2. No difference was found in access to transplant by availability of MELD in the database in the last two periods (2011–2016: HR 1.19 [95% CI 0.97, 1.45] *p* = 0.093; and 2017–2022: HR 1.08 [95% CI 0.88, 1.32] *p* = 0.475). Percentage of patients with available MELD per year is presented in Supplementary Table S3.

### Analysis of the Donor Pool

Donor characteristics are described in Supplementary Table S4. A steady increase in the number of donations has been seen in our study since 2014, coinciding with an expansion in the use of DCD livers (from 2.4% before 2011 to 15.8% after this date, *p* < 0.001). The COVID-19 pandemic explains the brisk drop in donations in 2020, now in recovery (Supplementary Figure S1). Men were more likely to receive a graft from a male donor (58.7%) while women received grafts from female donors more often (56.5%, *p* < 0.001 for the difference). Female donors were shorter than male donors (164.6 ± 11.8 vs. 168.2 ± 10.4 cm, *p* < 0.001). There were no differences in allocation of DCD or DBD livers by sex of the recipient, although female recipients received split livers more frequently (1.9% in female vs. 0.9% in male recipients, *p* < 0.001).

### Influence of Sex in Access to LT in Spain

**FIGURE 5:**
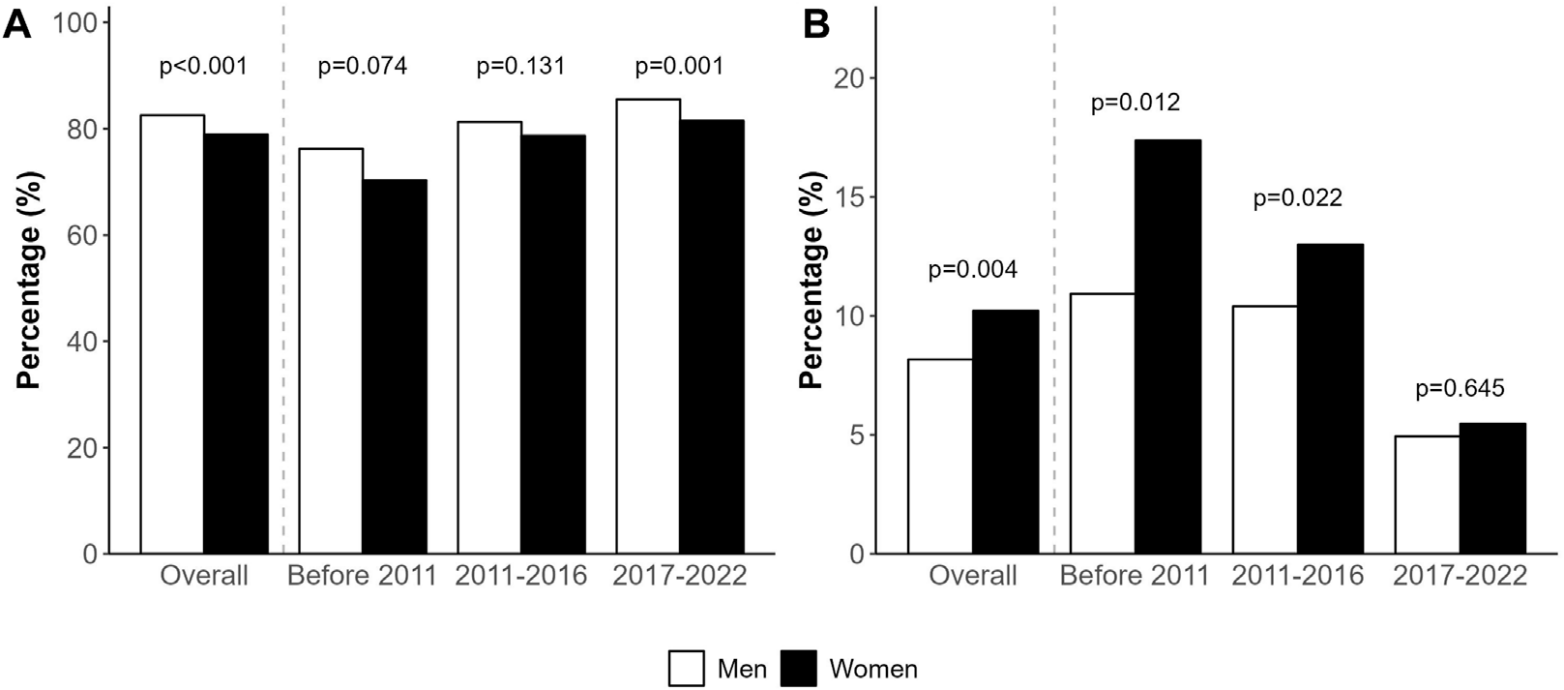
**(A)** Transplanted patients by sex, overall and by period. **(B)** Exclusion from the waiting list due to deterioration or death by sex, overall and by period.

Overall, fewer women received a LT (79% vs. 82%, *p* < 0.001) and a greater proportion were excluded (10% vs. 8%, *p* = 0.004) from the WL compared to men. Even though still present, these differences have decreased in recent years (Figure 5).

The overall probability of women undergoing LT was lower (OR 0.78, 95% CI 0.63, 0.97; *p* = 0.022) after adjusting for age, height, blood group and MELD score. These differences have attenuated in the last decade. After adjusting for recipient’s age, height and blood group, the probability of being transplanted was lower for women before 2011 (OR 0.68, 95% CI 0.49, 0.96; *p* = 0.026). In this first period, MELD data were scarce and could not be added to the model. However, in the last two periods, after adding MELD to the model, no significant differences in access to liver transplantation were found by sex (2011–2016: OR 0.82 [95% CI 0.60, 1.13] *p* = 0.216; and 2017–2022: OR 0.77 [95% CI 0.57, 1.05] *p* = 0.094). Time on the WL did not seem to influence the risk of women undergoing transplant (HR 0.95 [95% CI 0.90, 1.01] *p* = 0.093).

The risk of exclusion from the WL due to deterioration or death was higher for women after adjusting for age, height and blood group, although the result did not reach statistical significance (HR 1.17 [95% CI 0.99, 1.38] *p* = 0.060). After adding MELD to the model, differences were no longer present (HR 1.01 [95% CI 0.75, 1.36] *p* = 0.928). When analysed by period, this inequity has subsided over time. Before 2011 (MELD not included in the model), the risk of being excluded from the WL was higher for women (HR 1.49 [95% CI 0.99, 2.25] *p* = 0.054). In the second (2011–2016) and third (2017–2022) periods, including MELD in the analysis, HR were 0.93 [95% CI 0.64, 1.36] (*p* = 0.716), and 0.93 [95% CI 0.57, 1.51] (*p* = 0.769), respectively.

Overall, mean waiting times for women were longer (198.6 ± 338.9 days for women vs. 173.3 ± 285.5 for men, *p* < 0.001). Over the last two decades, waiting times have shortened for both sexes, but women still wait longer than men (Table 3). In particular, women with intermediate MELD scores [16–20] waited significantly longer than men with similar scores (Table 4). In this subgroup of women with intermediate MELD scores, despite longer waiting times, there was no significant difference in access to transplant (HR 1.10, 95% CI 0.82, 1.48; *p* = 0.534) or risk of being excluded from the WL for deterioration or death (HR 0.98, 95% CI 0.61, 1.57; *p* = 0.925).

**TABLE 3:** Time on waiting list by sex and period.

Period	Waiting time (days)		
	Men	Women	P^a^
2000–2010	408.4 ± 593.1	473.6 ± 693.7	0.078
2011–2016	186.5 ± 223.1	207.0 ± 251.0	**0.005**
2017–2022	88.7 ± 118.2	104.2 ± 148.2	**<0.001**

All results are expressed as mean ± SD. One-way ANOVA *p* < 0.001 for the comparison between periods both for men and for women.

^a^ Welch Two Sample t-test for the comparison between men and women.

The bold values represent p values that are significant statistically.

**TABLE 4:** Time on waiting list by sex in patients included since 2011 with available MELD.

MELD score	Waiting time (days)		
	Men	Women	P^a^
<16	160.0 ± 198.6	167.8 ± 206.8	0.455
16–20	183.3 ± 184.5	223.4 ± 234.5	**<0.001**
>20	100.4 ± 168.4	125.4 ± 220.1	0.129

All results are expressed as mean ± SD.

^a^ Welch Two Sample t-test for the comparison between men and women.

The bold values represent p values that are significant statistically.

Among patients with HCC, there were no differences in access to LT by sex (data not shown).

## Discussion

Our study presents national Spanish data on WL demographics over the last 20 years, confirming an aging population and a shift in aetiologies towards less viral hepatitis and more MASLD-related liver disease. Waiting times in our country have significantly decreased over time. Women were found to have lower access to transplant and a higher risk of exclusion due to worsening or death compared to men, although these differences have reduced in recent years, in parallel with shorter waiting times.

In our cohort, only 23% of patients on the WL were females. This percentage remained stable throughout the study period. Female representation in the Spanish WL is slightly lower than the 40% reported in the literature in other countries [1, 6]. Not only women were under-represented on the WL, but they were also less likely to receive a LT and had a higher risk of being excluded from the WL for being too sick for LT. This is in keeping with several US based-studies showing women to be at higher risk of death or drop-out on the WL and less likely to receive an organ [1, 24].

There is no published information as to the burden of decompensated cirrhosis in Spanish women, but data from a recent systematic analysis allows us to estimate a 40% prevalence of decompensated cirrhosis in Spanish women and 60% in men, similar to other regions of the world [25]. Yet only around 20% of women and 80% of men finally access LT waitlists in Spain. This difference with other series could be explained by the high number of HCC indications in our country (34%), compared, for instance, to the most recent OPTN report in the US showing that HCC was the primary diagnosis for 10.5% of waitlist candidates [26]. Indeed, HCC is more frequent in men (38% vs. 22% in our study, *p* < 0.001). A traditionally healthier lifestyle in women has translated into lower rates of alcohol-related liver disease, hepatitis C infection and HCC, although this might change in the future with the increase of MASLD in women. One important finding in our study is the decreasing rate of mortality and exclusion due to deterioration in our WL, both in males and females, with differences between sexes disappearing in recent years (Figure 5).

Several changes have occurred in the LT field over the last decade in Spain that help interpret our results. Firstly, public health interventions have resulted in a decrease in the number of patients listed for a LT. In particular, universal treatment of hepatitis C from 2015 has allowed our country to witness a decreased number of indications for LT associated with hepatitis C-related diseases [27], as depicted in Figure 3. This national plan to eradicate hepatitis C decreased the number of patients requiring a transplant, resulting in shorter waiting times a few years later (Figure 4) [27]. Secondly, Spain consistently reports the highest rates of deceased donation in the world (14,383 valid donors during our study period), based on the implementation of the so called “Spanish Model in Organ Donation and Transplantation” that has been well described in the literature [16, 28]. Over the last years, the implementation of innovative measures such as the standardization of intensive care to facilitate organ donation, the expansion of donor eligibility criteria and the incorporation of DCD (with the systematic use of normothermic regional perfusion) has further allowed to increase the availability of livers for clinical use [29]. In fact, the global percentage of DCD use in our study was 14.3%. The COVID-19 pandemic impacted significantly in donation rates and transplant programs, but this is now in recovery. Finally, MELD was progressively adopted in different regions of Spain since 2003 and became the allocation method of choice in the majority of the country from 2011.

Around the world, adoption of MELD derived systems as the preferred allocation policy translated into a decrease in global mortality on the WL [30, 31]. While implementation of MELD based systems in other countries was associated with a further reduction in rates of transplantation among women compared with the previous era (9% vs. 14% reduction rate in the pre vs. post MELD era) [3], we found the opposite (Figure 5A), with a growing number of women accessing transplant. The most accepted explanation for the sex-based difference in access to LT is the use of creatinine, which underestimates renal dysfunction in women because of their lower muscle mass [9–11, 32] and their smaller stature [13, 15]. Similar studies performed in North America show that differences between sexes in terms of transplantation, death or removal from list are small during the first months after listing but grow progressively after 1 year of waiting and remain stable after 3 years [10]. We found an association between a longer time on the WL in women and the risk of exclusion for worsening or death prior to 2011 (HR 1.49, 95% CI 0.99, 2.25; *p* = 0.054) that disappeared after this date (HR 1.11, 95% CI 0.92, 1.33; *p* = 0.277). As mentioned above, many changes occurred in the LT field after 2011, which makes it difficult to point to a single explanation for the observed improvement in sex-related inequities. In our particular scenario, for instance, where access to transplantation occurs in less than 6 months, patients listed with HCC may not gain enough points to reach the top of the list, which could minimize the differences between men and women. As previously noted, overall waiting times are very short, which probably contributes to women not being penalized with higher drop-out rates due to worsening or death despite longer waiting times than men, in particular those with intermediate MELD scores.

The main strength of our study is the use of a large national database including a large number of patients with long follow up. It is also one of the few works addressing access to LT by sex outside the United States. It has, however, some limitations. MELD data are incomplete, and although there does not seem to be any significant difference between patients with reported MELD and those without from 2011, there is a risk of measurement or information bias, and caution should be exerted when interpreting and extrapolating the results. The MELD system was progressively, but non-homogeneously, adopted in different regions of Spain since 2003 becoming the allocation method of choice in most of the country in 2011. However, the collection of this piece of information, despite its importance, is not mandatory in the current Registry. This, in addition to the retrospective nature of the study dating up to 20 years ago, explain the incomplete and fragmented MELD data (see Supplementary Table S3 for the evolution of MELD registration). The Spanish LT community should take this opportunity to engage in appropriate data collection, so that Registry studies can offer solid evidence as to how our excellent system performs. No other relevant predictors of WL mortality [33] have been explored, due to the retrospective nature of the study. Finally, there are, still nowadays, significant differences in WL times and donation rates between regions in Spain. However, we have described the global results of one of the most praised transplant systems in the world. Recently, MELD 3.0 was proposed as the official allocation policy in the United States [34]. Future studies in our setting where waiting times are short should address its usefulness.

In summary, even in countries with short waiting times, women wait longer and have a lower access to transplant and higher risk of exclusion from the WL. Policies directed at optimizing the whole LT network should be encouraged to guarantee a fair and equal access of all patients to this life saving resource.

## Data Availability

The raw data supporting the conclusion of this article will be made available by the authors, without undue reservation.

## Footnotes

1 Requests to access these datasets should be directed to www.ont.es.

## Abbreviations

AIH, autoimmune hepatitis; ANOVA, analysis of variance; CORE, Spanish Registry for Donation and Transplantation; DBD, donation after brain death; DCD, donation after cardiac death; HCC, hepatocellular carcinoma; HR, hazard ratio; Kg, kilograms; LD, living donation; LT, liver transplantation; MASLD, metabolic dysfunction-associated steatotic liver disease; MELD, model for end-stage liver disease; ONT, Organización Nacional de Trasplantes (National Transplant Organization); OR, odds ratio; RETH, Registro Español de Trasplante Hepático (National Spanish Liver Transplant Registry); US, United States; vs., versus; WL, waiting list.
