# Supplementary material for: Women Are Also Disadvantaged in Accessing Transplant Outside the United States: Analysis of the Spanish Liver Transplantation Registry
Source: Transpl Int. 2024 May 7;37:12732. doi: 10.3389/ti.2024.12732 (PMC11106452; doi:10.3389/ti.2024.12732)
Supplement: Supplementary file 1 [file Table1.DOCX]

| Variable | Included patients  (n=14385) | Excluded patients | | |
| --- | --- | --- | --- | --- |
|  |  | **Urgent^¥^**  **(n=1207)** | **Re-transplants**  **(n=305)** | **Combined**  **(n=80)** |
| Age (years) | 56.2 ± 8.7 | 48.0 ± 12.9^†^ | 52.0 ± 11.9^†^ | 52.1 ± 11.7^†^ |
| Weight (kg) | 77.3 ± 15.7 | 74.5 ± 16.2^†^ | 70.0 ± 14.7^†^ | 68.6 ± 16.4^†^ |
| Height (cm) | 168.4 ± 8.6 | 168.0 ± 8.6 | 167.4 ± 9.4 | 163.3 ± 10.4 |
| MELD^*^ | 16.6 ± 5.7 | 25.1 ± 7.3^†^ | 20.5 ± 6.9^†^ | 23.8 ± 4.5^†^ |
| Time on WL (days) | 179.0 ± 298.6 | 47.9 ± 130.8^†^ | 261.7 ± 490.4 | 221.6 ± 312.3 |
| Women | 3270 (22.7%) | 521 (43.2%)^†^ | 100 (32.8%)^†^ | 27 (33.8%)^†^ |

**Supplementary Table 1. Baseline demographics of the included and excluded patients.** Continuous variables are expressed as Mean ± SD; categorical variables are expressed as n (%). ^†^Welch Two Sample t-test: p<0.005 for comparison against included patients. ^¥^ Urgent transplants include acute and subacute indications. ^*^MELD data only available for 5475 patients.
