# Supplementary figures and images for: Women Are Also Disadvantaged in Accessing Transplant Outside the United States: Analysis of the Spanish Liver Transplantation Registry
Source: Transpl Int. 2024 May 7;37:12732. doi: 10.3389/ti.2024.12732 (PMC11106452; doi:10.3389/ti.2024.12732)

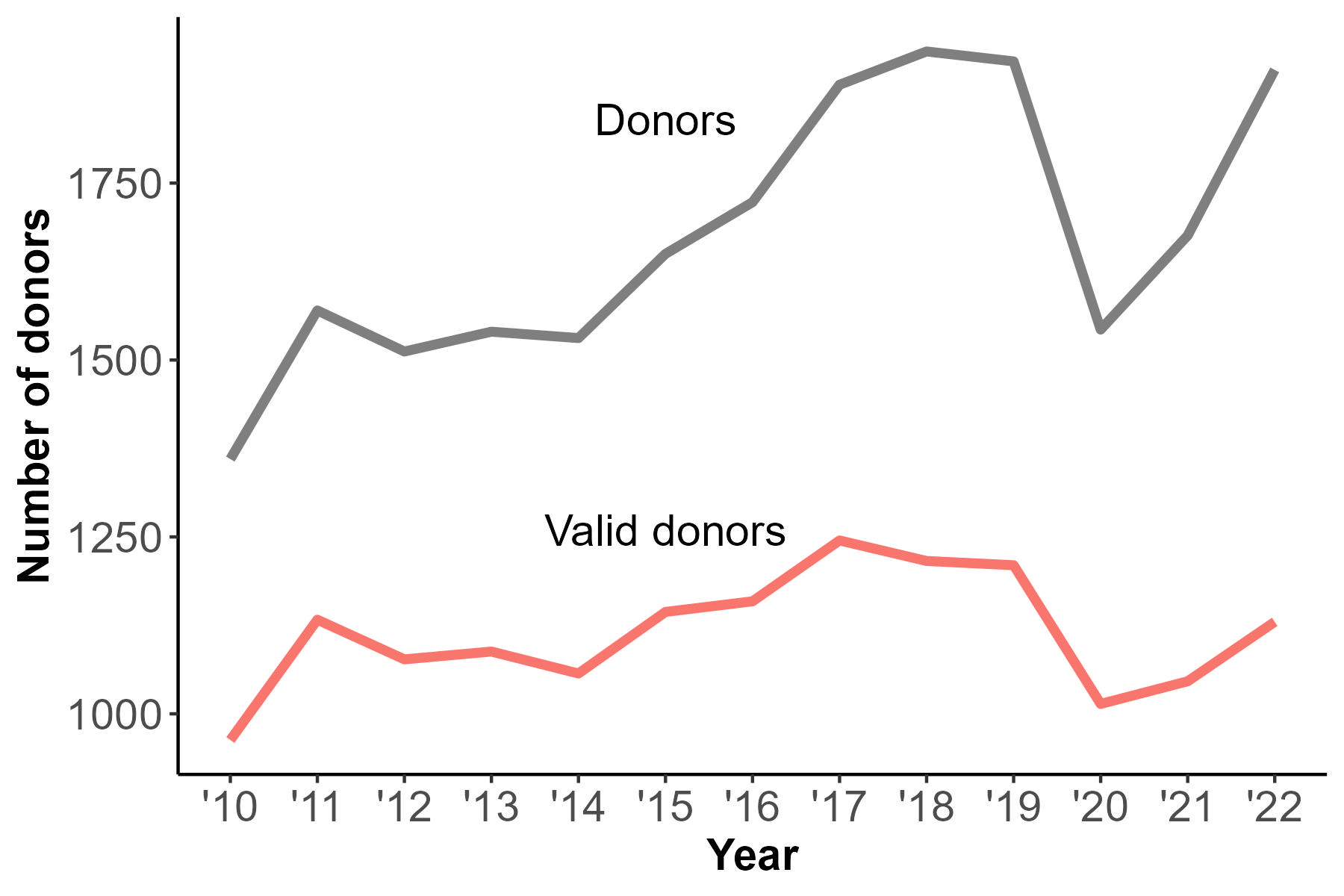

Supplement: Supplementary file 2 [file Image1.JPEG]
