# Supplementary material for: Women Are Also Disadvantaged in Accessing Transplant Outside the United States: Analysis of the Spanish Liver Transplantation Registry
Source: Transpl Int. 2024 May 7;37:12732. doi: 10.3389/ti.2024.12732 (PMC11106452; doi:10.3389/ti.2024.12732)
Supplement: Supplementary file 3 [file Table2.DOCX]

| Variable | Available MELD  (n=5350) | Unavailable MELD  (n=7249) | p^1^ |
| --- | --- | --- | --- |
| Age (years) | 56.5 ± 8.6 | 56.6 ± 8.7 | 0.278 |
| Weight (kg) | 77.7 ± 15.5 | 77.5 ± 16.0 | 0.321 |
| Height (cm) | 168.5 ± 8.7 | 168.4 ± 8.6 | 0.457 |
| Women | 1197 (22.4%) | 1638 (22.6%) | 0.768 |
| Blood group  · A  · O  · B  · AB | 2296 (42.9%)  2267 (42.4%)  560 (10.5%)  227 (4.2%) | 3420 (47.2%)  2838 (39.2%)  685 (9.4%)  307 (4.2%) | **<0.001** |
| Aetiology  · Alcohol  · Viral  · Cholestatic  · MASLD  · AIH  · Other | 2400 (44.9%)  1545 (28.9%)  314 (5.9%)  221 (4.1%)  117 (2.2%)  753 (14.0%) | 3156 (43.5%)  2116 (29.2%)  408 (5.6%)  261 (3.6%)  179 (2.5%)  1129 (15.6%) | 0.088 |
| HCC | 1932 (36.1%) | 2482 (34.2%) | **0.029** |

**Suplementary Table 2. Comparison of baseline demographics by MELD availability from 2011.** Continuous variables are expressed as Mean ± SD; categorical variables are expressed as n (%). ^1^ Welch Two Sample t-test for comparison between men and women (continuous variables); Pearson's Chi-squared test (categorical variables). MASLD: metabolic dysfunction-associated steatotic liver disease. AIH: autoimmune hepatitis. HCC: hepatocellular carcinoma.
