# Supplementary material for: Women Are Also Disadvantaged in Accessing Transplant Outside the United States: Analysis of the Spanish Liver Transplantation Registry
Source: Transpl Int. 2024 May 7;37:12732. doi: 10.3389/ti.2024.12732 (PMC11106452; doi:10.3389/ti.2024.12732)
Supplement: Supplementary file 4 [file Table3.DOCX]

| **Year** | **Available MELD**  (n = 5475) |
| --- | --- |
| 2022 | 456 (47.3%) |
| 2021 | 447 (45.9%) |
| 2020 | 391 (41.6%) |
| 2019 | 409 (38.3%) |
| 2018 | 377 (36.7%) |
| 2017 | 548 (55.7%) |
| 2016 | 520 (51.1%) |
| 2015 | 544 (46.3%) |
| 2014 | 430 (37.3%) |
| 2013 | 457 (40.2%) |
| 2012 | 434 (39.1%) |
| 2011 | 337 (32.1%) |
| 2010 | 76 (7.1%) |
| 2009 | 20 (4.0%) |
| 2008 | 12 (10.6%) |
| 2007 | 5 (10.2%) |
| 2006 | 9 (33.3%) |
| 2005 | 2 (15.4%) |
| 2004 or previous | 1 (11.1%) |

**Supplementary Table 3.** Number and percentage of patients with available MELD per year.
