# Supplementary material for: Women Are Also Disadvantaged in Accessing Transplant Outside the United States: Analysis of the Spanish Liver Transplantation Registry
Source: Transpl Int. 2024 May 7;37:12732. doi: 10.3389/ti.2024.12732 (PMC11106452; doi:10.3389/ti.2024.12732)
Supplement: Supplementary file 5 [file Table4.DOCX]

| Donor characteristic | All  (n=14383) | Recipient’s sex | | |
| --- | --- | --- | --- | --- |
|  |  | **Male**  **(n=11113)** | **Female**  **(n=3270)** | **p^1^** |
| Age (years) | 59.5 ± 16.0 | 59.8 ± 15.8 | 58.8 ± 16.8 | **0.013** |
| Weight (kg) | 75.4 ± 13.8 | 76.9 ± 13.6 | 70.1 ± 13.0 | **<0.001** |
| Height (cm) | 167.4 ± 10.8 | 168.2 ± 10.4 | 164.6 ± 11.8 | **<0.001** |
| Graft type |  |  |  | 0.175 |
| ·DBD | 9922 (84.9%) | 7716 (84.8%) | 2206 (85.3%) |  |
| ·DCD | 1671 (14.3%) | 1320 (14.5%) | 351 (13.4%) |  |
| ·LD | 53 (0.5%) | 38 (0.4%) | 15 (0.6%) |  |
| ·Domino | 40 (0.3%) | 27 (0.3%) | 13 (0.5%) |  |

**Supplementary Table 4. Donor demographics, overall and by recipient’s sex.** Continuous variables are expressed as Mean ± SD; categorical variables are expressed as n (%). ^1^ Welch Two Sample t-test for comparison between men and women (continuous variables); Pearson's Chi-squared test (categorical variables). DBD: donation after brain death. DCD: donation after cardiac death. LD: living donation.
